# The use of complex structures with a word class change in Inuktitut child-directed speech

**DOI:** 10.3389/fpsyg.2022.971395

**Published:** 2022-10-07

**Authors:** Olga Alice Johnson, Shanley E. M. Allen

**Affiliations:** Center for Cognitive Science, Faculty of Social Science, University of Kaiserslautern, Kaiserslautern, Germany

**Keywords:** child-directed speech, morphological simplification, polysynthesis, Inuktitut, first language acquisition, nominalization, noun incorporation, variation sets

## Abstract

Caregivers typically use a simplified mode of the language – child-directed speech (CDS) – when addressing young children. In this study, we investigate the use of complex morphological structures with a word class change within a single word in Inuktitut CDS. Inuktitut is a polysynthetic agglutinative language of the Inuit–Yupik–Unangan language family spoken in arctic Quebec, which allows more than 10 morphemes per word and in which the meaning of an entire sentence can be expressed in one word. Clearly, such a complex morphological system presents special challenges for young children, which raises the question of whether caregivers shape their CDS in ways that facilitate acquisition. Using the data from mothers addressing eight Inuktitut-speaking children aged 0;11 to 3;6, we investigated whether the frequency and complexity of polysynthetic structures in CDS are dependent on the stage of the children’s linguistic development. The results demonstrate that the number and morphological complexity of the structures with a word class change increased as the children developed linguistically. The variety of nominalizers and verbalizers – the key components of such structures – also increased through the stages and were used in variation sets, which help children acquire morphological items by providing examples of use of the same morpheme in morphologically contrasting environments. These results show the presence of morphological simplification in Inuktitut CDS and demonstrate that such simplification is fine-tuned, i.e., that mothers are sensitive to their children’s level of linguistic development.

## Introduction

The great complexity of human language presents a number of challenges for children’s learning. While their ability to master their native language relatively quickly with no formal instruction is remarkable, it is critical that children get substantial linguistic input during the first several years of their life (Friedmann and Rusou, 2015). This leads to one of the central questions in language development: what is the nature of linguistic input necessary for first language acquisition? To answer this question, much research has focused on investigating a special simplified mode of speech used by caregivers, presumably to facilitate acquisition – child-directed speech (CDS)^1^.

In those languages that show evidence of this simplified mode, the simplification affects numerous aspects of caregivers’ speech (e.g., McLeod, 1993; Green et al., 2010; Kunert et al., 2011). Effects in phonology, syntax and the lexicon have been studied extensively in the past decades, demonstrating notable differences between CDS and adult-directed speech (ADS) (e.g., Snow, 1972, 1995; Gallaway and Richards, 1994). When compared to adult-directed speech, CDS is usually slower, has higher pitch and a greater pitch range. It is characterized by syllable-lengthening, longer pauses, exaggerated intonation and stress, as well as shorter and simpler utterances, a restricted range of vocabulary, and a prevalence of concrete words. The morphological aspects of CDS, however, have received much less attention.

Gaining more knowledge about morphology in CDS is crucial given the number of morphologically rich languages around the world and the challenges that morphology presents in their acquisition. Polysynthetic languages in particular offer much opportunity to explore the morphological aspects of CDS due to their complex morphological structure. However, to date, very few studies have been conducted on the morphology of CDS in polysynthetic languages (e.g., Crago and Allen, 2001; Lester et al., 2022; Johnson et al., in press). In the present study, we provide insight into how the morphosyntax of CDS is adjusted based on children’s linguistic ability in Inuktitut – a polysynthetic language spoken in arctic Canada. We use the CDS data from the Inuktitut-speaking mothers of eight typically developing children aged 0;11 to 3;6^2^ to investigate a particular morphological feature of Inuktitut – polysynthetic structures in which the word class changes within a single word – and the key components of those structures – nominalizers and verbalizers. We ask whether the mothers make such structures more accessible for young children by adjusting the frequency of use and the complexity of such structures in CDS according to the children’s stage of linguistic development, and/or by providing clues that help children identify the relevant morphemes in the stream of speech.

## Background and motivation for the study

Many studies propose that CDS in some form exists across all cultures, despite substantial differences in style and frequency of use (Fernald, 1992; Bryant and Barrett, 2007; Saxton, 2009; Piazza et al., 2017). Studies have shown multiple differences between the speech addressed to child and adult interlocutors, providing clear evidence that CDS is a separate speech mode, distinct from ADS (e.g., Snow, 1972; Ferguson, 1978; Ringler, 1981; Gallaway and Richards, 1994; Foulkes et al., 2005). Adults adapt their speech addressed to children in numerous ways including structured repetition (Küntay and Slobin, 1996; Lester et al., 2022), exaggerated articulation (Lindblom, 1990; Minjung and Stoel-Gammon, 2005; Green et al., 2010) and prosody (Fernald and Simon, 1984; McLeod, 1993), syntactic and lexical simplification (Fernald and Morikawa, 1993; Kunert et al., 2011), and a large emphasis on interaction (Hoff and Naigles, 2002). Numerous studies also show that CDS is preferred by children (Fernald, 1985; Schachner and Hannon, 2011; Masapollo et al., 2016). When it comes to the morphology of CDS, however, there is still a distinct shortage of literature.

### Morphology of child-directed speech

The existing studies on the morphology of CDS show two main patterns: that caregivers tend to structure their CDS in a way that promotes acquisition of various morphological features, and that the input children receive from caregivers influences their production with regards to morphology. The first pattern is evidenced by studies across a number of structures in which difficult morphology is highlighted or dehighlighted in CDS. For example, Kempe et al. (2001) showed that caregivers adapted diminutives differently across three languages to facilitate transparency of nominal gender. Note that diminutives make nominal gender less transparent in German since all diminutives take neuter case and thus obscure the case of the base noun [e.g., *der Hund* ‘the(M) dog,’ *das Hündchen* ‘the(N) doggie’]. However, they make nominal gender more salient in Spanish and Russian since the gender of the base noun is maintained and made more salient through the diminutive ending that also reflects gender [e.g., *el perro* ‘the(M) dog,’ *el perrito* ‘the(M) doggie(M)’]. Interestingly, Kempe et al. (2001) found that German CDS contained a lower proportion of diminutives as compared to Russian and Spanish CDS. They concluded that, by using fewer diminutives, German-speaking caregivers de-emphasized a linguistic feature of German that would complicate the acquisition of nominal gender, while Russian- and Spanish-speaking caregivers made that morphological feature more salient by using more diminutives.

Ravid et al. (2008) found similar evidence with noun plurals across four languages. While being transparent and predictable from a semantic point of view, inflectional systems in many languages are opaque and irregular when it comes to morphology, thus presenting serious challenges for young children. The authors demonstrated that the inflectional system in CDS in German, Dutch, Danish, and Hebrew made plural suffixes much more predictable and regular as compared to ADS and that the regularities were made prominent in all the languages investigated. For example, in German, feminine nouns can become plural by attaching several suffixes: the most productive *-en* (*Farm-en* ‘farms’), productive *-s* (*Farm-s* ‘farms’) and *-n* (*Vase-n* ‘vases’), and unproductive *-e* (*Bräut-e* ‘brides’) and a zero suffix (*Mütter* ‘mothers’). In German CDS, however, feminine plurals with *-en* appeared even more frequently than in ADS, both in the sense of distributional asymmetry and overall productivity, which made the most productive feminine plural suffix more salient for children. Similarly, in Dutch, nouns ending in an obstruent take –*en* as a suffix in 97.6% of cases in CDS, while in only 93.0% of the cases in ADS. The authors concluded that caregivers structured their CDS in a way that supported their children’s acquisition of the nominal plural markers.

A study on obviative demonstratives in North East Cree shows the important role CDS plays in revealing “morphosyntactic facets of the language that may not be as frequent or readily apparent in adult-level speech” (Henke and Brittain, 2022, p. 89). Obviation is a characteristic feature of Algonquian languages that distinguishes between two types of third person. In a given clause, there is one ‘proximate’ third person referent – i.e., the center of the discourse, while all others are obligatorily designated ‘obviative.’ After analyzing more than 25 h of video recordings of one adult addressing three children (between the ages of 2;01 and 5;10), the authors conclude that structures that are particularly frequent in CDS require the use of demonstratives. Demonstratives, in their turn, play a crucial role in encoding obviation, especially in possessive constructions. Demonstratives help disambiguate equational constructions that lack nouns and verbs; often serve as more precise grammatical markers than nouns; and, finally, they overtly encode the obviative status of possessees in cases when nouns and verbs lack such marking. Although the main goal of the study was to emphasize the role of CDS in providing valuable material for linguistic description, it also clearly demonstrates how the properties of CDS promote acquisition by overtly encoding some grammatical information that would not be encoded by verbs and nouns in the same context.

A separate group of studies emphasizes the role of variation sets for making morphology more salient. Lester et al. (2022) broadly describes a variation set as a tightly clustered set of partially repetitive utterances that are linked by interactional context. In analytic languages, such as English, a variation set involves a repetition of a word or a syntactic structure in a variety of syntactic contexts. In Example (1), first introduced by Küntay and Slobin (1996, p. 267), the verb *see* and the question ‘Who did we see?’ are repeated in different syntactic environments.

**Table:** 

(1)	Verb ***see*** and question ‘*Who did we see?*’ in a variation set
	*Who did we* ***see*** when we went out shopping today?
	*Who did we* ***see****?*
	*Who did we* ***see*** in the store*?*
	*Who did we* ***see*** today*?*
	When we went out shopping, *who did we* ***see****?*
	(Father addressing his child, 2;3)

Therefore, by using variation sets in CDS, the caregiver, consciously or subconsciously, helps a child acquire certain syntactic structures and/or lexical or morphological items by providing repetition in combination with variability.

Variation sets have been well documented for lexical and syntactic phenomena in English (e.g., Küntay and Slobin, 1996; Waterfall, 2006; Brodsky et al., 2007). Recently, however, studies have examined their use for morphologically rich languages as well. For example, Lester et al. (2022) examined child-surrounding speech in eight typologically maximally diverse languages and found variation sets in all of them (Chintang, English, Inuktitut, Japanese, Russian, Sesotho, Turkish, and Yucatec). While these results suggest that the use of variation sets in CDS is universal, the frequency trends for different languages varied considerably. In some languages, the proportion of variation sets started to decrease as the child got older. In morphologically rich languages (i.e., Chintang, Turkish, and Inuktitut), however, the prevalence of variation sets increased over time, which suggests that the caregivers actively employ this speech register to promote acquisition of morphology as the children develop linguistically.

Another study on variation sets in Dene and Qaqet showed that adults modify their speech when addressing young children in order to make difficult morphemes more salient and, therefore, to facilitate their children’s acquisition (Hellwig and Jung, 2020). For example, a negation morpheme in Dene, which is normally a fused enclitic, is used by a mother in a variation set that first presents the combined expression (verb + negative enclitic) and then – immediately – presents the verb and the enclitic separately. This allows the child, firstly, to identify the stem and the particle as separate morphemes, and, secondly, to access the negation morpheme’s underlying form, which, when used in combined expressions, undergoes considerable morphophonological changes. In Qaqet, where all nouns are preceded by a vowel-final article, some root-initial consonants can be lenited (‘softened’), which makes identification of the underlying form impossible. In ADS, the underlying form surfaces very rarely. In CDS, however, those normally obligatory initial articles are regularly omitted, which pushes the initial consonant of the noun root into initial position and makes it surface. Both examples illustrate how CDS provides a natural context for underlying forms to surface, thus making them easier to identify as morphemes and to acquire. The authors suggest that the patterns of simplification and clarification used in CDS resemble those used by native speakers when they explain the structure of their language to a researcher.

The second main pattern in studies on morphology in CDS highlights the alignment in morphology production between CDS and child speech. A cross-linguistic study on nominal inflections showed that children produced noun plurals (types and tokens) with a frequency that closely matched that of the input they received from their caregivers (Ravid et al., 2008). The authors demonstrated that the ratio of noun plurals in CDS (about 20% of all noun types and 10% of noun tokens) was “closely echoed” by the ratio of noun plurals in the children’s speech (about 16% plural types and 7% plural tokens). The aspects that make this study particularly relevant to our research are, firstly, its concentration on the core morphology – the most productive patterns and the most prototypical members of the morphological system of a language – and, secondly, presenting a systematic longitudinal analysis of spontaneous speech data.

Another longitudinal study shows that the dominant verb forms correspond closely between CDS and child speech (Veneziano and Parisse, 2010). Previous studies of early verb form acquisition showed that young children produce individual verbs in one form only, although not necessarily the same form across verbs. For example, in English, a child may at first use the verb ‘to close’ only in the form of *closed* and the verb ‘to open’ only in the form of *open*. This is true for languages with both limited and rich inflectional morphology. The study with two French-speaking caregiver-child pairs (children aged 1;3–2;2 and 1;7–2;3) showed that, firstly, the caregivers used the majority of verbs in one morphophonological form only when addressing their children, and, secondly, that 64% of the verb forms produced by one child and 80% of verb forms produced by the other child corresponded with the dominant verb forms in their caregivers’ speech. They also found that the verb forms produced by the children were further reinforced in conversational contingencies in interactions between the children and their caregivers, demonstrating the caregivers’ sensitivity to the children’s production and their effort to adjust CDS to their children’s level.

Similarly, a study with four Inuktitut-speaking children (1;11–2;1) showed that they used verbal inflections with 96% of verb roots on average, which corresponded to the input they received from their mothers who used the obligatory verbal inflections in 99% of cases in their CDS (Crago and Allen, 2001). Since Inuktitut is a null-subject language, verbal inflections play an important role in expressing person and number of the referent. Children acquiring languages that do not permit null subjects typically go through a stage of optional infinitive production – a period during which they produce both fully inflected verbs and verbs with no inflections. The results of this study suggest that typically developing Inuktitut-speaking children do not go through an optional infinitive stage and that Inuktitut-speaking mothers use considerably more overt markers of finiteness as compared to English-speaking mothers, which could be a contributing factor in such early acquisition of verbal morphemes by Inuktitut-speaking children.

A counterpoint to the study by Crago and Allen (2001) is a study with two Sesotho-speaking children (both 2;1) and their caregivers, which also demonstrated that the properties of input shape the acquisition of morphology (Ziesler and Demuth, 1995). While the omission of grammatical morphology is typical for all children in the early stages of language acquisition (e.g., Bloom, 1970), the explanation for this phenomenon is still debated. The authors hypothesized that the omission of the noun class prefixes they observed in the Sesotho-speaking children’s data resulted from patterns in CDS. Results showed that over 70% of the input to both children consisted of nominal forms that either did not require a prefix or where the prefix was dropped, which supported the authors’ hypothesis. Particularly relevant to the present study are findings that support the hypothesis that the prefix dropping in CDS correlated with children’s grammatical development. While both children were the same age, child A received and produced a higher percentage of passives and relatives, as well as fewer dropped prefix forms, as compared to child B. The authors proposed that this was due to the fact that, at that point in the acquisition process, A might be focusing more on syntactic structure, and B on nominal structure, thus suggesting that the caregivers (including siblings) simplified the morphology of the speech when addressing the children in accordance with the stages of the children’s linguistic development.

In all the studies just reviewed, caregivers shaped their speech directed to children in a way that provided multiple clues for the use of morphology. They promoted their children’s acquisition of difficult morphological features in many ways: by highlighting the word forms and categories that help acquisition and de-highlighting those that make the morphology more opaque, by making complex morphological features more predictable and regular as compared to ADS, by making complex morphology more salient by using variation sets, by structuring CDS in a way that illuminates the distributional aspects of the morphological system, by using morphological simplification and clarification similar to those used when a native speaker explains the morphological system of their language to a researcher, and by simplifying morphology according to the level of children’s linguistic development. Two of these – the use of variation sets and a fine-tuned morphological simplification of CDS – served as motivation for the approach we take in the current study. In line with many studies on morphology of CDS, we used a longitudinal analysis of data from spontaneous speech to show that Inuktitut-speaking caregivers shape their use of morphology in a way that promotes acquisition of complex structures. In contrast to the previous research, this study focuses on the use of more complex morphological structures in CDS and shows that their frequency and complexity increase gradually in accordance with children’s level of linguistic development.

### Inuktitut morphology

Eastern Canadian Inuktitut is one of the four groups of Inuit languages that belong to the Inuit–Yupik–Unangan language family (Dorais, 2010). This group of languages is spoken by some 34,000 speakers and is further divided into dialects and subdialects. In the present study, we investigate the Tarramiut dialect, which is spoken by some 3,000 speakers in the Hudson Strait area of arctic Quebec (Allen, 1996). For the sake of simplicity, in this paper we will refer to it as ‘Inuktitut.’

Inuktitut has three word classes (noun, verb, and other), more than 1,000 obligatory nominal and verbal inflections, and more than 400 optional word-internal morphemes (e.g., tense, aspect, negation, passive, and causative). Its polysynthetic structure allows more than ten morphemes per word, with an average word length of 3.72 morphemes, as compared to 1.68 morphemes per word on average in English (Greenberg, 1960). Example (2) illustrates the morphological complexity of Inuktitut by demonstrating how its polysynthetic agglutinative structure allows expressing the meaning of an entire sentence in one word^3^:

**Table:** 

(2)	*Illujuaraalummuulaursimannginamalittauq*.
	illu-juaq-aluk-mut-uq-lauq-sima-nngit-gama-li-ttauq
	house-big-EMPH-ALL.SG-*go*-PAST-PERF-NEG-
	CTG.1sS-but-also
	‘But also, because I never went to the really big house.’
	(Dorais, 1988, p. 8)

Two morphological features of Inuktitut are particularly relevant for the present study: noun stem incorporation within the verbal complex and verb-to-noun shifting. Noun incorporation is a structure in which both the verb and the object argument are contained in the same word. The word begins as a noun and then changes into a verb through addition of a bound verbal suffix, which we refer to as a ‘verbalizer.’ An example of noun incorporation with the verbalizer *si* ‘buy’ is in (3).

**Table:** 

(3)	*Mukulsingittu*.
	immukuluk-***si***-nngit-juq
	milk-*buy*-NEG-PAR.3sS
	‘He didn’t buy the milk.’
	(Jini’s mother addressing Jini’s sister, 13)

Shifting of a verb to a noun is also common through addition of a bound nominal suffix that we refer to as a ‘nominalizer.’ An example of a verb shifting to a noun with the nominalizer *juq* ‘that which’ is in (4). Both these structures can occur in the same word, meaning that, in Inuktitut, a word can change its class several times within a single word.

**Table:** 

(4)	*Hantatugulu Saali*?
	haanta-***juq***-guluk saali
	ride.honda-*that.which*-EMPH.PEJ charlie
	‘Is little Charlie riding a Honda?’ [lit. ‘is Charlie a little one
	who is riding a Honda?’]
	(Elijah’ mother addressing Elijah, 3;0)

Example (5) demonstrates a typical utterance in which the word class changes twice in the first word (noun-to-verb-to-noun) through affixation of the verbalizer *qaq* ‘have’ and the nominalizer *juq* ‘that which’, and once in the second word (verb-to-noun).

**Table:** 

(5)	*Pitaqangitualummi iputiqanguatuq.*
	pi-ta-***qaq***-nngit-***juq***-aluk-mik iputi-***qaq***-nnguaq-juq
	thing-possession-*have*-NEG-*that.which*-EMPH-MOD.SG paddle-*have*-play.at-PAR.3sS
	‘He’s pretending to paddle with nothing.’ [lit. ‘he is pretending to have a paddle
	which does not have anything’]
	(Elijah’s mother addressing Elijah, 2;8)

### The present study

It is clear that the complex morphology of Inuktitut presents special challenges for acquisition: children have to learn to identify and correctly use morphemes in long and morphologically complex words, while the polysynthetic nature of Inuktitut means that the individual forms are not repeated very frequently (Stoll et al., 2012). This raises the question of whether the input that children get from their caregivers is shaped in a way that would help them in that task. The few existing studies on the morphology of Inuktitut CDS suggest that this is indeed the case (Crago and Allen, 2001; Lester et al., 2022).

The present study extends this research by examining polysynthetic structures where the word class changes within a word in Inuktitut CDS (as shown in Examples 2–5). We approached the study in two ways. First, we asked whether the frequency and complexity of such structures in CDS are dependent on the stage of the children’s linguistic development. Based on previous literature, we hypothesized that the morphology of CDS would be simpler for children at lower stages of linguistic development, and, thus, that the number of complex structures and their morphological complexity would increase as the children advance linguistically. Specifically, we predicted that mothers would use structures with a word class change more frequently as their children progress linguistically and that the complexity of such structures in CDS would increase from one word class change in earlier stages to several word class changes within a single word in later stages. This would demonstrate that mothers simplify their CDS morphology and that this simplification is fine-tuned (Sokolov, 1993; Snow, 1995).

Second, we asked how mothers use the key components of the structures with a word class change – verbalizers and nominalizers – in each stage and across the stages. In particular, we focused on the frequency of use of each component, and whether and how they are used in variation sets (Küntay and Slobin, 1996). In morphologically rich languages such as Inuktitut, variation sets are often centered around morphemes rather than words. When producing a variation set in Inuktitut, a caregiver would typically use a certain morpheme in morphologically contrasting environments in successive pairs of utterances as illustrated in (6).

**Table:** 

(6)	Verbalizer ***u*** ‘be’ and question word *kina* ‘who’ in a
	variation set
(a)	*Kinamuun*?
	kina-mut
	who-ALL.SG
	‘By whom?’
(b)	*Kinaummaan*?
	kina-***u***-mmat
	who-*be*-CTG.3sS
	‘What’s his name?
(c)	*Kinautsunii*?
	kina-***u***-tsuni
	who-*be*-CTM.4sS?
	‘What’s his name?’
	(Paul’s mother addressing Paul, 2;6)

In example (6), *u* ‘be’ is used where a word changes its class once: noun (nominal question word)-to-verb. It is embedded in two different morphological environments: immediately following the nominal question word *kina* ‘who’; and immediately preceding two verbal inflections – *mmat* ‘CTG.3sS’ and *tsuni* ‘CTM.4sS.’ In the first utterance (*Kinamuun*?), ***u*** is absent while the question word *kina* is present; this provides a clue that ***u*** and *kina* are not parts of the same morpheme.

In polysynthetic languages, the use of variation sets by the caregiver helps the child identify relevant morphemes rather than perceiving them as single units. Repetition, which is a part of any variation set, promotes learning of the morphemes. And since a variation set shows a variation of the contexts in which a particular morpheme can appear, it helps the child to start using that morpheme productively rather than as part of a fixed form. Use of variation sets in the data suggests that the mothers, consciously or subconsciously, alter their speech when addressing their children, presumably to facilitate their acquisition of morphology.

The rest of the article is structured as follows. Section “Participants and data” presents information on the participants and data. Section “Trends in use of polysynthetic structures across the stages” presents the analyses of the use of polysynthetic structures across the stages, including structures where the word class changes both once and more than once within a word. In Section “The use of verbalizers and nominalizers,” we investigate the use of the main components of such structures – verbalizers and nominalizers – in each stage and across the stages, focusing on their frequencies and the use of the more frequent nominalizers and verbalizers in variation sets. Finally, Section “Discussion” concludes the article with discussion of the use of complex polysynthetic structures in CDS and directions for further research.

## Participants and data

### Participants

Participants were eight Inuktitut-speaking mothers and their children aged 0;11–3;6. All the participants lived in small communities in arctic Quebec. Five of the mothers (aged from early to late 20s) were biological mothers of the children, while the other three (aged from late 40s to late 50s) were adoptive mothers (two of them were the children’s grandmothers), as shown in Table 1.

**TABLE 1:** Participant information (child ages in years; months.days).

Mother	Age	Relationship to child	Child	Age	People in household	Generations in household
1	57	Adoptive (grandmother)	Jini (f)	0;1.28–2;0.20	4	4
2	20	Biological	Tumasi (m)	1;9.11–2;9.30	13	3
3	52	Adoptive	Lucasi (m)	1;7.30–2;8.15	6	2
4	21	Biological	Sarah (f)	1;3.26–2;4.6	3	2
5	29	Biological	Paul (m)	2;6.6–3;3.2	4	2
6	48	Adoptive (grandmother)	Elijah (m)	2;0.11–2;9.5	8	3
7	23	Biological	Lizzie (f)	2;6.2–3;2.26	4	2
8	29	Biological	Louisa (f)	2;9.16–3;6.12	6	2

### Data collection

The data come from two sets of video recordings of spontaneous naturalistic interactions between mothers and their children (Crago, 1988; Allen, 1996)^4^. The amount of data varied across children and sessions. In the first set of recordings (mothers 1–4 in Table 1), the interactions between four mothers and their children (aged 0;11–1;8 at onset) were recorded in two communities of fewer than 400 inhabitants. The data were collected four times throughout a year at three-and-a-half-month intervals, resulting in about 80 h of video recordings (about 5 h per child per data collection point). For the second set of recordings (mothers 5–8 in Table 1), four mothers and their children (aged 2;0–2;10 at onset) were recorded in one community of about 250 inhabitants. The data were collected nine times over 9 months at 1-month intervals, resulting in about 130 h of video recordings. In both studies, recordings were typically done in two to four sessions of 30–120 min over a 1-week period – referred to collectively as a ‘data collection point’ – rather than in one session of 4–5 h. During all sessions, other family members and the children’s friends were often present and participated in the interactions. However, only the mothers’ speech addressing the target children was analyzed in the present study.

### Data preparation

About half the recordings from each data collection point were selected for transcription, on the basis of recording quality and talkativeness of the child and mother. Thus, portions of recordings were excluded if there was too much background noise, if the recording was faulty, if the child was crying or fussy or silent for extended periods, if there were a large number of visitors such that the child was less in focus or transcription was too difficult, etc. The utterances spoken by and to each target child in the selected recordings were transcribed and translated into English by native speakers of Inuktitut, following the CHAT format of the CHILDES project (MacWhinney, 2000).

#### Data coding

The morphemes were then identified and glossed in ATOM – a text editor that allows a semi-automated procedure of coding morphemes in each word. First, each utterance is entered separately, accompanied by its translation into English, information on the speaker, the addressee, and the time, as well as non-verbal information (if available) to provide context for the utterance. A coder then highlights a part of a word, and all matching morphemes and their meanings from the Inuktitut-English dictionary are displayed. When the appropriate morpheme is selected, it is added to the separate morpheme tier (%mor). The process continues until a morphological code for each utterance is created. Example (7) shows an utterance produced by a mother (MOT) addressing a target child (CH1) at 34 min and 39 s from the beginning of the session.

**Table:** 

(7)	MOT: Piijaliruk.
	%eng: Take it apart.
	%mor: NR|PLEON|pi&thing+VZ| ijaq&remove+VV|
	liq&POL+VI|guk&IMP_2sS_3sO
	%tim: 00:34:39
	%add: CH1
	(Elijah’s mother addressing Elijah, 2;2)

In (7), the utterance consists of one word, which consists of four morphemes. The morpheme tier shows that the word starts as a noun (NR), then changes into a verb with a verbalizer (VZ), which is followed by a verbal suffix (VV) and a verbal inflection (VI). Thus, morpheme tiers allow finding structures with a word class change by searching for their main components – verbalizers and nominalizers.

#### Data by stage

The data were then divided into six groups based on the children’s stage of linguistic development (Johnson et al., In press). Due to the fact that children’s linguistic abilities do not develop at the same rate, mean length of utterance in morphemes (MLUm) was chosen over chronological age as an indicator of linguistic development (Allen and Dench, 2015). The stages were determined by children’s MLUm for each file. Table 2 shows the distribution of data (the number of child-directed utterances produced by the mothers) in the six stages. The table also specifies the children’s MLUm that was used to define each stage, the children’s mean age and age range, and the number of mothers whose data were used in each stage. Each stage contains different amounts of data from several mothers at more than one recording session^5^.

**TABLE 2:** Data statistics by stage.

	Stage 1	Stage 2	Stage 3	Stage 4	Stage 5	Stage 6
Children’s MLUm	1.0–1.5	1.5–2.0	2.0– 2.5	2.5–3.0	3.0–3.5	>3.5
Children’s mean age	1;7	2;1	2;7	2;7	2;11	2;9
Children’s age range	0;11–2;3	1;8–2;8	2;0–3;1	2;1–2;10	2;3–3;5	2;4–3;1
Mothers	4	3	4	3	4	2
Mothers’ utterances	2550	1043	1764	2958	1045	1885

We further divided the data in each stage into ‘data collection points’ (DCPs), i.e., units that include all the data from one mother that were recorded within 1 month, which resulted in about 4–5 h of recorded data per DCP. Each stage has a different number of DCPs: eight in Stage 1, five in Stage 2, nine in Stage 3, eleven in Stage 4, six in Stage 5, and seven in Stage 6. Table 3 provides an example of data preparation for Stage 2, which includes five DCPs (each with 2–3 recording sessions) and data from three mothers. The third column shows the total number of polysynthetic structures where the word class changes from verb (V→) within a single word produced by the mother in each recording session, with a total for each DCP in the fourth column. The seventh column provides the mean number of the V→ structures per utterance per DCP, which was calculated by dividing the number of the V→ structures in each DCP (column 6) by the number of utterances in that DCP (column 5). Finally, the last column shows the means per 100 utterances per DCP.

**TABLE 3:** The number of structures that start as a verb (V→) and change its class within a word in CDS in Stage 2, per data collection point (DCP).

Data files by DCP	Child’s age	V→	V→ per DCP	Number of utterances	Number of utterances per DCP	Mean V→ per utterance per DCP	Mean V→ per 100 utterances per DCP
Jini’s mother 17	1;08.05	2	4	112	225	0.0622	6.22
Jini’s mother 18	1;08.05	2		91			
Jini’s mother 19	1;08.05	0		22			
Jini’s mother 110	2;00.19	5	17	106	332	0.0481	4.81
Jini’s mother 111	2;00.20	5		105			
Jini’s mother 112	2;00.20	7		121			
Lucasi’s mother 310	2;08.12	5	6	68	124	0.0241	2.41
Lucasi’s mother 312	2;08.12	1		56			
Sarah’s mother 47	1;11.07	1	13	59	177	0.0338	3.38
Sarah’s mother 48	1;11.07	5		18			
Sarah’s mother 49	1;11.07	7		100			
Sarah’s mother 411	2;04.05	6	8	114	185	0.0054	0.54
Sarah’s mother 412	2;04.05	2		71			

## Trends in use of polysynthetic structures across the stages

Our first research question asks what trends are evident in the use of polysynthetic structures in CDS across the children’s early stages of development. We specifically focused on polysynthetic structures where the word class changes within a word through use of nominalizers and/or verbalizers. We hypothesized that caregivers would use fewer polysynthetic structures in earlier stages as compared to later stages and that the complexity of such structures in CDS would increase from Stage 1 to Stage 6. We looked at the number of structures used overall at each stage, with particular emphasis on structures with increasing amounts of complexity as measured by the number and type of word class changes within a given word.

### Structures where the word class changes within a word

We examined the structures in CDS where the word class changes within a word. To find such structures, we searched for nominalizers and verbalizers using CLAN (MacWhinney and Snow, 1990). Utterances that comprised immediate and exact self-repetitions were counted as one instance. Since each stage has a different number of recordings and the recordings are not equal in length, we calculated the mean number of such structures per 100 utterances. We also differentiated data according to the type of word class change (N → V, V → N, etc.) as well as the number of word class changes within one word. In the CDS data, we found polysynthetic structures with up to four word class changes. Data are summarized in Table 4.

**TABLE 4:** Use of structures with word class change in CDS across developmental stages (N/no. per 100 utterances).

	Stage 1	Stage 2	Stage 3	Stage 4	Stage 5	Stage 6
N → V	72/3	49/5	146/8	172/6	84/8	177/9
V → N	52/2	25/2	100/6	178/6	65/6	161/8
N → V → N	3/<1	2/<1	27/1	24/1	8/1	43/2
V → N → V	16/1	15/1	51/3	85/3	25/2	94/5
N → V → N → V	2/<1	3/<1	4/<1	16/<1	3/<1	22/1
V → N → V → N			5/<1	6/<1	1/<1	7/<1
N → V → N → V → N				1/<1		3/<1
V → N → V → N → V				3/<1		1/<1

We begin by discussing the simplest instances of polysynthetic structures – those that contain only a single change of word class: N → V and V → N. Not surprisingly, these are the most frequent in the data, and also clearly increase in use across the six stages of development that we examined. Changes from noun to verb (8) are most common, tripling in use across our stages from three per 100 utterances at Stage 1 to nine per 100 utterances at Stage 6. Example (8) shows how a noun changes into a verb with the verbalizer *it* ‘be.’

**Table:** 

(8)	*Qariamilluti*.
	qariaq-mi-***it***-lutit
	bedroom-LOC.SG-*be*-ICM.2sS
	‘Stay in the bedroom.’
	(Lizzie’s mother addressing Lizzie, 2;6)

Changes from verb to noun (9) are almost as common, quadrupling in use from two per 100 utterances at Stage 1 to eight per 100 utterances at Stage 6. Example (9) shows how a verb becomes a noun with the nominalizer *juq* ‘one which.’

**Table:** 

(9)	*Aahaaturulu*.
	aahaaq-***juq***-guluk
	hurt-*one.which*-EMPH.PEJ
	‘Little one is in pain.’ [lit. ‘one who is in pain’]
	(Jini’s mother addressing Jini, 2;0)

Cases where a word class changes twice within a single word (N → V → N and V → N → V) are also relatively frequent. While the structures with the noun-to-verb-to-noun change were only used three times (<1 per 100 utterances) in Stage 1, their number reached one per 100 utterances in Stage 3, stayed at that level in Stages 4 and 5, and then doubled in Stage 6. Example (10) demonstrates how a noun changes into a verb and back to a noun using the verbalizer *mitiq* ‘cover with’ and the nominalizer *juq* ‘one which.’

**Table:** 

(10)	*Aputimitirnatualu*.
	aputi-***mitiq***-naq-***juq***-aluk
	snow-*cover.with*-CAUS-*one.which*-EMPH
	‘Thing that gets covered with snow.’
	(Elijah’s mother addressing Elijah, 2;9)

The number of structures with the verb-to-noun-to-verb change increases five-fold from Stage 1 to Stage 6: from one to five per 100 utterances. Example (11) shows how a verb changes to a noun with the nominalizer *juq* ‘one which’ and then back to a verb with the verbalizer *u* ‘be.’

**Table:** 

(11)	*Ijukkalaurtualuuvutit*.
	ijukka-lauq-***juq***-aluk-***u***-vutit
	fall-PAST-*one.which*-EMPH-*be*-IND.2sS
	‘You are one who fell.’
	(Elijah’s mother addressing Elijah, 2;9)

We now move to the next level of complexity, where the word class change occurs three times within a single word: N → V → N → V and V → N → V → N. The number of structures with the noun-to-verb-to-noun-to-verb change increased from only two instances (less than one per 100 utterances) in Stage 1 to two per 100 utterances in Stage 6. Example (12) demonstrates how a noun changes into a verb using the verbalizer *qaq* ‘have,’ then to a noun with the nominalizer *juq* ‘one which,’ and then to a verb using the verbalizer *u* ‘be.’

**Table:** 

(12)	*Ataatatsiaqangituulirit.*
	ataatatsiaq-***qaq***-nngit-***juq***-***u***-liq-git
	grandfather-*have*-NEG-*that.which-be*-POL-IMP.2sS
	‘You’re the only one who’s not going to have a grandfather.’
	(Louisa’s mother addressing Louisa, 3;0)

While the structures with the verb-to-noun-to-verb-to-noun change appear in every stage starting from Stage 3, they are only used 19 times across Stages 3 – 6 (<1 per 100 utterances in each stage). Example (13) shows how a verb changes to a noun with the nominalizer *vik* ‘place,’ then to a verb with the verbalizer *u* ‘be,’ and back to a noun with the nominalizer *suuq* ‘habitually.’

**Table:** 

(13)	*Mauna turquiviusuuq kiinaujarni.*
	ma-uuna turquC-i-***vik-u-suuq*** kiinaujaq-nik
	here-VIA put.away-ANTP-*place-be-HAB* money-MOD.PL
	‘Here is where you stuff the coins.’
	(Elijah’s mother addressing Elijah, 2;9)

Finally, the rarest structures in the data are the ones where the word class change occurs four times within a single word: N → V → N → V → N and V → N → V → N → V. The structure with the noun-to-verb-to-noun-to-verb-to-noun change first appears in Stage 4 (used once) and then reappeared in Stage 6 (used three times) – i.e., <1 per 100 utterances in each stage. Example (14) demonstrates how a noun changes into a verb using the verbalizer *mitiq* ‘cover with,’ then to a noun with the nominalizer *juq* ‘one which,’ then to a verb using the verbalizer *u* ‘be,’ and back to a noun with the nominalizer *juq* ‘one which.’

**Table:** 

(14)	*Nipittaamitirijualuulirtui!*
	nipittaaq-***mitiq***-gi-***juq***-aluk-***u***-liq-***juq***-it
	sticky.thing-*cover.with*-again-*that.which*-EMPH-*be*-PRES-
	*that.which*-ABS.PL
	‘They [the drinks] are getting it [the side-table] sticky!’ [lit.
	‘they are ones which are again covering it with stickiness’]
	(Elijah’s mother addressing Elijah, 2;2)

The structures with the verb-to-noun-to-verb-to-noun-to-verb change appeared in the data starting from Stage 4 and were used four times: three times in Stage 4 and once in Stage 6 (<1 per 100 utterances in each stage). Example (15) shows how a verb changes to a noun with the nominalizer *juq* ‘one which,’ then to a verb with the verbalizer *u* ‘be,’ to a noun with the nominalizer *juq* ‘one which,’ and, finally, back to a verb with the verbalizer *u* ‘be.’

**Table:** 

(15)	*Uiviittuuqattatuviniulautu?*
	uiviit-***juq-u***-qattaq-***juq***-viniq-***u***-lauq-jutit?
	annoy-*that.which-be*-HAB-*that.which*-former-*be*-PAST-
	PAR.2sS
	‘Didn’t you pester?’ [lit. ‘were you one who habitually was
	one who pestered?’]
	(Elijah’s mother addressing Elijah, 2;9)

**FIGURE 1:**
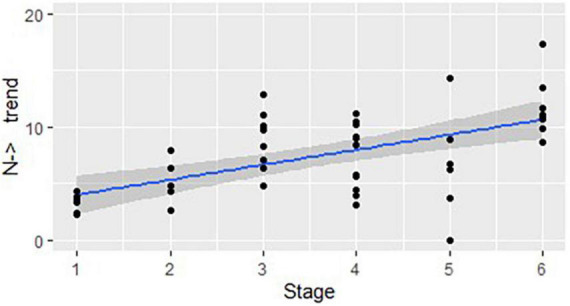
The developmental trend in structures that start as a noun and change their class: correlation between the stages of linguistic development and the mean number of structures used in each DCP (represented by dots), per 100 utterances.

**FIGURE 2:**
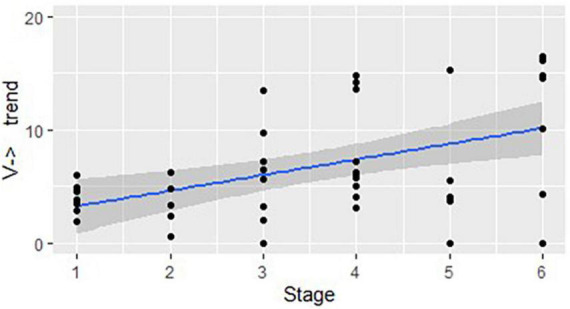
The developmental trend in structures that start as a verb and change their class: correlation between the stages of linguistic development and the mean number of structures used in each DCP (represented by dots), per 100 utterances.

For statistical comparison of use of the structures across stages, we began by calculating the number of structures that start as nouns (N→) and those that start as verbs (V→) and then undergo at least one word class change, by DCP, for each stage (see Section “Data by stage”). Then the means per utterance per DCP were calculated. To do that, we divided the number of N→ structures and V→ structures in each DCP by the number of utterances in that DCP. We then multiplied each mean by 100 to find the number of polysynthetic structures per 100 utterances for each DCP in each stage. A Pearson correlation test was run for both sets to see the trends. Figure 1 shows that the overall use of the structures that start as a noun and change their class (N→) within a single word is positively correlated with the stages of linguistic development: *r*(44) = 0.43, *p* < 0.01.^6^ Figure 2 shows a similar trend for all structures that start as a verb and then change their class (V→): *r*(44) = 0.58, *p* < 0.01.

**FIGURE 3:**
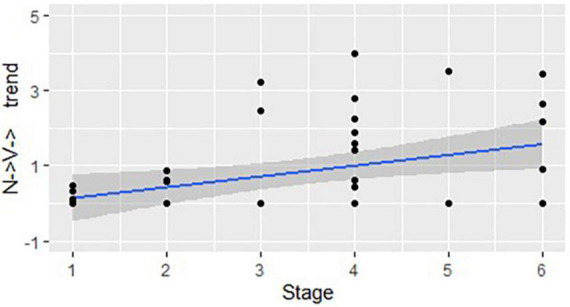
The developmental trend in structures that start as a noun and change their class more than once: correlation between the stages of linguistic development and the mean number of structures used in each DCP (represented by dots), per 100 utterances.

**FIGURE 4:**
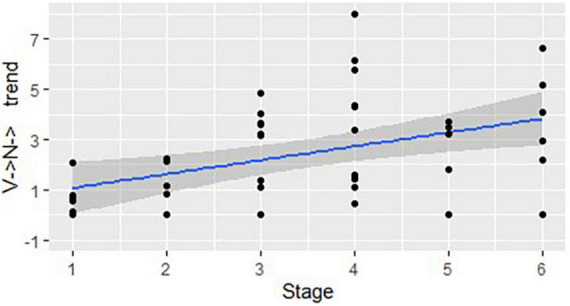
The developmental trend in structures that start as a verb and change their class more than once: correlation between the stages of linguistic development and the mean number of structures used in each DCP (represented by dots), per 100 utterances.

We then repeated the same procedure for the structures where the word class changes more than once within a word. Figure 3 demonstrates a positive correlation between the stages and the structures that start as a noun and change their class more than once (N → V→): *r*(44) = 0.48, *p* < 0.01. Figure 4 shows a positive correlation between the use of the structures that start as a verb and change their word class more than once (V → N→) and the stages of linguistic development: *r*(44) = 0.48, *p* < 0.01.

To summarize, the results demonstrate that the mothers gradually increase the number of complex polysynthetic structures in their CDS and that the complexity of the structures they use grows as the children progress through the stages of linguistic development. The structures with one class change (N → V and V → N) are used the most across the data. However, their percentage decreases gradually from 85% in Stage 1 to 66% in Stage 6, as they give space to the growing number of structures of higher complexity. The number of structures with two word class switches (N → V → N and V → N → V) increases from 13 to 27%, making this type of structure the second most frequent in the data. Although the usage of the structures with three word class changes (N → V → N → V and V → N → V → N) is relatively low, they appear in all stages, and their frequency increases the most among all types of structures: from 1% in Stage 1 to 6% in Stage 6. Finally, the structures of the highest complexity (N → V → N → V → N and V → N → V → N → V) only appear in later stages (Stages 4 and 6) and comprise less than 1% of all structures with a word class change used in the CDS data.

## The use of verbalizers and nominalizers

We then looked at the key components of complex polysynthetic structures – verbalizers and nominalizers – separately. We counted them using CLAN and calculated their frequencies by stage and across the CDS data. Based on the results presented in Section “Trends in use of polysynthetic structures across the stages,” we hypothesized that the frequency of use and the variety of nominalizers and verbalizers in CDS would also increase with the stages of the children’s linguistic development.

### Verbalizers

A total of 30 different verbalizers were used in CDS data. Table 5 shows that the variety of verbalizers (N types) grew from 10 in Stage 1 to 24 in Stage 6, with the highest number of different verbalizers (27) used in Stage 4. The highest number of different verbalizers per 100 utterances (N types) was used in Stage 5. The number of tokens (N tokens) per 100 utterances increased by 4.3 times: from six in Stage 1 to 26 in Stage 6.

**TABLE 5:** The number of verbalizers (N type/token) and the number of verbalizers per 100 utterances (N type/token), by stage.

	Stage 1	Stage 2	Stage 3	Stage 4	Stage 5	Stage 6
N (type/token)	10/162	15/131	19/333	27/545	20/200	24/492
N (type/token) per 100 utterances	<1/6	1/12	1/19	1/19	2/19	1/26

Some verbalizers were used more frequently than others across the stages (Table 6). The verbalizer *u* ‘be’ comprised 30.5% of all the verbalizers used in CDS, followed by *it* ‘be’ at 25.8%, *uq* ‘arrive at’ at 8.6% and *qaq* ‘have’ at 7.2%. The most infrequently used verbalizers – *iq* ‘remove’ and *guk* ‘craving’ – each appeared in the data only once (0.05%), in Stage 1 and in Stage 4, respectively. As shown in Table 6, all the verbalizers used in CDS, except one (*niaq* ‘hunt’), first appeared in the data before Stage 5. Verbalizers *aq* ‘go by way of,’ *guq* ‘become,’ *iq* ‘remove,’ *it* ‘be,’ *liuq* ‘make,’ *qaq* ‘have,’ *taq* ‘fetch,’ *tuq* ‘consume,’ *u* ‘be,’ and *uq* ‘arrive at’ were used starting from Stage 1; while *niaq* ‘hunt’ appeared for the first time in Stage 6. Only six of the verbalizers used in CDS did not appear in Stage 6. The mothers added 1 to 7 verbalizers per stage, with the exception of Stage 5, in which no new nominalizers were used.

**TABLE 6:** Frequencies of verbalizers as a percentage of the total number of verbalizers used in the data, by stage.

	Stage 1	Stage 2	Stage 3	Stage 4	Stage 5	Stage 6
*aq* ‘go by way of’	13.4	7.8	7.0	4.6	5.5	6.1
*gi* ‘have as’		2.3	2.7	1.7	1.5	3.5
*guk* ‘craving’				0.2		
*guq* ‘become’	1.2			0.2	2.5	1.4
*ijaq* ‘remove’		0.8		0.7	0.5	2.0
*iq* ‘remove’	0.6					
*iruq* ‘become without’			0.3	0.2		0.4
*it* ‘be’	25.0	21.8	25.8	29.9	32.0	20.4
*it* ‘be without’				0.2		0.2
*laq* ‘remove’		3.1		0.4	0.5	
*liaq* ‘go to’			4.5	1.5	2.5	1.8
*liri* ‘work with’		4.6	1.2	1.3	3.0	1.8
*liuq* ‘make’	0.6	2.3	1.2	1.7	0.5	3.2
*mitiq* ‘cover with’			1.2	0.7	1.5	0.8
*ngaaq* ‘rather’		0.8	0.3	1.3	2.0	0.4
*ngau* ‘toward’			0.3		0.5	
*ngu* ‘feel sick’				0.4	1.0	
*niaq* ‘hunt’						0.8
*nnguq* ‘become’			0.6	0.7	1.0	0.8
*qaq* ‘have’	6.2	4.7	5.4	8.1	6.5	8.5
*si* ‘buy’			0.3	0.5		0.2
*siuq* ‘look for’			0.9	0.2	1.0	0.2
*taaq* ‘acquire’		3.1	1.2	2.4	1.0	1.2
*taq* ‘fetch’	0.6	0.8		0.2		0.2
*tuq* ‘consume’	5.6	7.0	8.8	2.9	4.0	3.9
*tuq* ‘play’				0.7		
*tuq* ‘ride’				0.5		0.8
*tuu* ‘exclusivity’		0.8	0.3	0.7		0.8
*u* ‘be’	24.6	31.4	26.7	32.5	26.0	34.1
*uq* ‘arrive at’	22.2	9.3	11.2	5.7	7.0	6.2

### Nominalizers

A total of 12 different nominalizers were used in CDS data. Table 7 shows that the variety (N types) of nominalizers grew from seven in Stage 1 to eleven in Stage 6. Both types and tokens per 100 utterances grew through the stages. The total number of nominalizers (N tokens) per 100 utterances increased by 6.6 times: from three in Stage 1 to 20 in Stage 6.

**TABLE 7:** The number of nominalizers (N type/token) and the number of nominalizers per 100 utterances (N type/token), by stage.

	Stage 1	Stage 2	Stage 3	Stage 4	Stage 5	Stage 6
N (type/token)	7/82	8/53	10/214	11/391	8/113	11/378
N (type/token) per 100 utterances	<1/3	1/5	1/12	<1/13	1/11	1/20

Some nominalizers were used more frequently than others across the stages (Table 8). The nominalizer *juq* ‘that which’ comprised 70.1% of all the nominalizers used in CDS, followed by *suuq* ‘habitually’ at 10.2% and *jaq* ‘passive’ at 9.6%. The most infrequently used nominalizers were *niqpaq* ‘superlative’ and *niq* ‘gerund,’ which only appeared in the data twice (0.16%) and three times (0.24%), respectively. As shown in Table 8, all the nominalizers used in CDS first appeared in the data before Stage 6. The nominalizers *jaq* ‘passive,’ *juq* ‘that which,’ *ji* ‘agentive,’ *qati* ‘companion,’ *suuq* ‘habitually,’ *siti* ‘expert,’ and *uti* ‘item used for’ were used starting from Stage 1. The nominalizers *niq* ‘gerund’ and *niqpaq* ‘superlative’ appeared in the data much later – in Stages 4 and 5, respectively. Only one of the nominalizers used overall in CDS did not appear in Stage 6. The mothers added 1 to 2 nominalizers per stage.

**TABLE 8:** Frequencies of nominalizers as a proportion of the total number of nominalizers used in the data, by stage.

	Stage 1	Stage 2	Stage 3	Stage 4	Stage 5	Stage 6
*gaq* ‘passive’			0.4	0.3	0.9	0.5
*jaq* ‘passive’	9.5	5.8	8.9	9.0	12.4	10.3
*ji* ‘agentive’	2.4		0.4	1.3		1.8
*juq* ‘that which’	76.2	65.4	74.2	67.6	68.2	70.2
*niq* ‘gerund’				0.5	0.0	0.3
*niqpaq* ‘superlative’					1.8	
*niqsaq* ‘comparative’			0.4	0.3	2.6	0.5
*qati* ‘companion’	4.8	1.9	1.0	0.5		0.5
*siti* ‘expert’	2.4	3.8	1.0	1.3		0.3
*suuq* ‘habitually’	3.6	15.4	7.6	13.8	8.0	9.5
*uti* ‘item used for’	1.1	5.8	4.7	4.1	2.6	5.3
*vik* ‘place’		1.9	1.4	1.3	3.5	0.8

To summarize, the results we obtained demonstrate that, when using complex morphological structures in CDS, the Inuktitut-speaking mothers increase the variety of their key elements – verbalizers and nominalizers – as well as their frequency of use, as their children progress linguistically. The increase appears to be in accordance with the stages of the children’s linguistic development.

### The use of verbalizers and nominalizers in variation sets

Based on the previous literature (Brodsky et al., 2007; Lester et al., 2022), we hypothesized that, in order to make nominalizers and verbalizers easier to identify and to remember, the mothers would use at least some of the nominalizers and verbalizers in variation sets. The fact that the same morpheme is repeated several times in a short time span makes variation sets an excellent tool for acquisition as repetition by itself facilitates learning and retention. Variation sets, however, provide more than repetition. The main feature of variation sets – a morphological overlap – makes the repeated morpheme more salient within long and complex polysynthetic structures and, therefore, further assists their acquisition.

The existing literature offers several approaches to what type of partially repetitive utterances can be considered a variation set (e.g., Waterfall, 2006; Brodsky et al., 2007; Grigonytė and Björkenstam, 2016; Wirén et al., 2016; Lester et al., 2022). In the present study, we rely on the approach developed by Brodsky et al. (2007), slightly adapted for the use in morphologically rich languages and for the purposes of the study. By this definition, a variation set is a sequence of utterances with a lexical or morphological overlap of one or more elements in successive pairs of utterances (e.g., first–second and second–third), where a particular nominalizer or verbalizer occurs with either different roots or suffix(es), and/or different inflections.

While an exhaustive search for all variation sets containing polysynthetic structures was beyond the scope of the present study, we manually identified numerous instances of variation sets containing some of the more frequent nominalizers and verbalizers used in the CDS. Complex polysynthetic structures with the most common nominalizer *juq* and the most common verbalizer *u* were used by the mothers in variation sets in all stages. Some of the other nominalizers and verbalizers were also used in variation sets in different stages, but not consistently. Variation sets in the data involved structures of different complexity: from one to four word class changes. We discovered three types of variation sets in the data that make the repeated verbalizers and nominalizers more salient. While all types were used across the stages, the first two patterns were more predominant in earlier stages and the third pattern was mostly used in the later ones. In (16) through (19), we provide several examples of variation sets of different types.

In the first type, a verbalizer/nominalizer is preceded by the same morpheme but followed by different ones. In (16), for example, the morpheme immediately preceding the verbalizer *tuq* ‘consume’ stays the same – the nominal root *qajuq* ‘soup.’ However, the following morphemes vary: in (16b) *tuq* is followed by the verbal affix *liq* ‘politeness,’ and in (16c) by the verbal affix *lauq* ‘politeness.’ In (16a), *tuq* is the final morpheme, which also help to identify the morpheme’s boundaries. In this variation set, the verbalizer *tuq* is used in structures where a word changes its class once (noun-to-verb).

**Table:** 

(16)	Verbalizer *tuq* ‘consume’ in a variation set in Stage 2.
(a)	*Qajurtuu atii*.
	qajuq-***tuq*** atii
	soup-*consume* initiate
	‘We’re having soup. Come on.’
(b)	*Qajurtulirit.*
	qajuq-***tuq***-liq-git
	soup-*consume*-POL-IMP.2sS
	‘Eat your soup.’
(c)	*Qajurtulaurit*.
	qajuq-***tuq***-lauq-git
	soup-*consume*-POL-IMP.2sS
	‘Eat your soup.’
	(Sarah’s mother addressing Sarah, 1;11, Stage 2)

In the second type, a verbalizer/nominalizer is preceded by different morphemes but followed by the same one. In (17), the most common nominalizer *juq* ‘that which’ is always followed by the same modifier *guluk* ‘emphatic pejorative.’ However, the preceding morphemes vary: in utterances (17a) and (17b), *juq* is preceded by the verbal affix *luaq* ‘very much,’ while in (17c) it directly follows the verbal root *miki* ‘be small.’ In this variation set, the nominalizer *juq* is used in structures with both one word class change (verb-to-noun) and two class changes (verb-to-noun-to-verb).

**Table:** 

(17)	Nominalizer *juq* ‘that which’ in a variation set in Stage 1.
(a)	*Qingaruluit mikijuatjukuluraluk*.
	qingaq-guluk-it miki-luaq-***juq***-guluk-aluk
	nose-EMPH.PEJ-ABS.2Ssg be.small-very.much-*that.which*-EMPH.PEJ-EMPH
	‘Your nose is very small.’ [lit. ‘your nose is one which is very small’]
(b)	*Mikijuatjukuluuk ukua*.
	miki-luaq-***juq***-guluk uku-a
	be.small-very.much-*that.which*-EMPH.PEJ here-ABS.DUPL
	‘These two are very small.’ [lit. ‘these two are ones which are very small’]
(c)	*Takugulugi mikijuruluutsutik*.
	taku-guluk-git miki-***juq***-guluk-u-tsutik
	see-EMPH.PEJ-IMP.2sS be.small-*that.which*-EMPH.PEJ-be-CTM.4dpS.
	‘Look, they’re so small.’ [lit. ‘they are ones which are small’]
	(Sarah’s mother addressing Sarah, 1;7)

In the third type, a verbalizer/nominalizer is both preceded and followed by different morphemes. In (18), the verbalizer *uq* ‘arrive at’ is immediately preceded by two morphemes: the nominal inflections *mut* ‘allative/Singular’ in (18a–c) and *kkut* ‘vialis/Singular’ in (18d). Immediately following *uq* come four morphemes: the verbal affixes *langa* ‘future’ in (18a), *gasuaq* ‘try’ in (18b), and *qattaq* ‘habitually’ in (18c), and the nominalizer *juq* ‘that which’ in (18d). In this variation set, the verbalizer *uq* is used in structures where a word changes its class once (noun-to-verb) and twice (noun-to-verb-to-noun).

**Table:** 

(18)	Verbalizer *uq* ‘arrive at’ in a variation set in Stage 6.
(a)	*Imarmulangamiju.*
	imaq-mut-***uq***-langa-mi-juq
	water-ALL.SG-*arrive.at*-FUT-also-PAR.3sS
	‘It will go in to the water’. [talking about hippopotamus on
	TV]
(b)	*Imarmurasuaqurtualuuguna*.
	imaq-mut-***uq***-gasuaq-qquuq-juq-aluk-u-na
	water-ALL.SG-*arrive.at*-try-probably-that.which-EMPH-
	here-ABS.SG
	‘It might try go in the water now.’ [talking about
	hippopotamus on TV.]
(c)	*Nanualu takulaujai*… *imarmuqattasuni*.
	nanuq-aluk taku-lauq-jait imaq-mut-***uq***-qattaq-tsuni
	polar.bear-EMPH see-PAST-PAR.2sS.3sO water-ALL.SG-*arrive.at*-HAB-CTM.4sS
	‘You saw the polar bear, it went to the water.’
(d)	*Takugi imakutukallaalu*.
	taku-git imaq-kkut-***uq***-juq-kallaq-aluk
	see-IMP.2sS water-VIA.SG-*arrive.at*-that.which-ENDR-EMPH
	‘Look at him going through water.’
	(Elijah’s mother addressing Elijah, 2;6)

In example (19), the nominalizer *suuq* ‘habitually’ is used in three types of structures: where the word class changes once (verb-to-noun), where it changes twice (verb-to-noun-to-verb), and where it changes four times (verb-to-noun-to-verb-to-noun-to-verb). A variety of morphemes immediately precedes *suuq*: the verbal affix *ji* ‘antipassive’ in (a) and (c); the verbal affix *jau* ‘passive’ in (b) and (d); and the verbal root *la* ‘say’ in (d). The two morphemes immediately following the nominalizer *suuq* are the verbalizer *u* ‘be’ in (a) and (b) and the nominal affix *aluk* ‘emphatic’ in (c) and (d). In (d), *suuq* is used twice, and in the second case it is the final morpheme of a word, which provides an additional clue that *suuq* is a separate morpheme.

**Table:** 

(19)	Nominalizer *suuq* ‘habitually’ in a variation set in Stage 6.
(a)	*Aniqujisugunngitualummata inuit*.
	ani-qu-ji-***suuq***-u-nngit-juq-aluk-u-mmata inuk-it
	go.out-want-ANTP-*HAB*-be-NEG-that.which-EMPH-be-CTG.3pS person-
	ABS.PL
	‘We, Inuit, never tell people to get out!’ [it’s impolite]
(b)	*Aniqujaausugunngimata aniqujigiaqanngimata*.
	ani-qu-jau-***suuq***-u-nngit-mmata ani-qu-ji-giaqaq-nngit-mmata
	go.out-want-PASS-*HAB*-be-NEG-CTG.3pS go.out-want-ANTP-must-NEG-
	CTG.3pS
	‘No one likes to be told to get out; therefore, you shouldn’t.’
(c)	*Aniqujauqattalaqtutit aniqujisuuraaluutuaruvit*.
	ani-qu-jau-qattaq-laaq-jutit ani-qu-ji-***suuq***-aluk-u-tuaq-guvit
	go.out-want-PASS-HAB-FUT-PAR.2sS go.out-want-ANTP-*HAB*-EMPH-be-
	as.soon.as-CND.2sS
	‘If you tell people to get out, they will do the same thing.’
(d)	*Aniliriit lasuuraluit aniqujausuut*.
	ani-liq-git la-***suuq***-aluk-it ani-qu-jau-***suuq***
	go.out-POL-IMP.2sS say-*HAB*-EMPH-ABS.PL go.out-want-PASS-*HAB*
	‘People who tell others to get out, usually end up getting told to go out.’
	(Elijah’s mother addressing Elijah, 2;7)

To summarize, these examples illustrate how variation sets provide a point of stability (a repeated morpheme) in slightly changing conditions (surrounding morphemes) facilitating the child’s acquisition of the morphological structure of Inuktitut. The presence of variation sets in the CDS data suggests that the mothers, whether consciously or subconsciously, alter their speech to create a better learning environment for their children.

## Discussion

In this study, we investigated whether and how Inuktitut-speaking mothers simplify their morphology when addressing children in order to facilitate their acquisition of morphemes and polysynthetic structures. The complexity of Inuktitut morphosyntactic structure presents many challenges for children. However, some studies show that Inuktitut-speaking children break into the structure at a young age: for example, children around the age of 2 years use obligatory verbal inflections in 96% of cases (Crago and Allen, 2001). The question, thus, arises if certain aspects of the input they receive facilitate their prompt acquisition.

While answering this question, we were both following in the steps of the previous research and developing our own approach. Analyzing previous work on CDS morphology, three main aspects can be distinguished. Firstly, it mainly focused on one morphological feature – inflections, as they can be found in both morphologically rich languages and in many of those with limited morphology, thus providing an opportunity for crosslinguistic comparison (e.g., Ravid et al., 2008). The current study, on the other hand, investigated a new type of structure – a more sophisticated aspect of morphology as compared to inflections – polysynthetic structures in which a word class changes (up to four times) within a single word. Secondly, many of the previous studies looked at the ways caregivers make a particular morphological phenomenon clearer for a child (e.g., Kempe et al., 2001; Hellwig and Jung, 2020), including the use of variation sets. We adopted this approach when investigating the key elements of structures with a word class change – nominalizers and verbalizers.

Finally, most of the previous studies concentrate on how caregivers adjust their speech when addressing children of a certain age or a certain stage of linguistic/cognitive development, but do not trace the development of such adjustments over time. In this regard, two studies stand out among other work on CDS morphology: the study by Lester et al. (2022) demonstrated how the change in frequency of variation sets in CDS over time is dependent on the morphological complexity of a language, while Ziesler and Demuth (1995) suggested correlation between the level of morphological simplification in CDS and children’s grammatical development. In our study, we extended this approach by looking at morphological complexity of CDS and at the use of variation sets as children went through the first six stages of linguistic development.

We hypothesized that the frequency and complexity of polysynthetic structures in CDS are dependent on the stage of the children’s linguistic development. Due to our focus on the gradual change of CDS morphology, we chose the children’s stage of linguistic development rather than their age as a predictor of the level of morphological complexity, as children do not acquire language at the same speed and may occasionally experience temporary regress. The results we obtained show that the morphological complexity of the structures with a word class change in CDS increases as the children develop linguistically. Both the number of structures where a word class change happens within a word and the number of structures where it happens more than once showed a significant increase from Stage 1 to Stage 6. The variety of nominalizers and verbalizers – the key components of complex polysynthetic structures – and their number per 100 utterances also increased through the stages. Mothers increased their variety by adding one to two new nominalizers and one to seven new verbalizers per stage. These results not only show the presence of morphological simplification in Inuktitut CDS but also demonstrate that such simplification is fine-tuned – in other words, that mothers are sensitive to their children’s level of linguistic development. We also found that nominalizers and verbalizers were used by the mothers in variation sets in all stages, which would help children acquire morphological items by providing examples of use of the same morpheme in morphologically contrasting environments.

Considering that variation sets make a repeated morpheme more salient, their presence in the CDS data is in line with the previous studies that showed caregivers making complex morphological features more salient for a child (Kempe et al., 2001; Ravid et al., 2008). Our results also support the findings of Lester et al. (2022), which showed that the use of variation sets in morphologically rich languages does not decrease as children grow older. While Lester et al. (2022) demonstrated that the prevalence of variation sets in CDS increased over time for languages with relatively more complex morphology (Inuktitut^7^, Chintang, and Turkish), their study took a strictly computational approach. Such an approach is very important for discovering broad patterns of variation set use across a large data set, but their work did not include examples from the data or discussion about the strategies of variation set use. We expand on those results by looking at what kinds of variation sets are present, how they are used, and how they reflect on morphological complexity of CDS in Inuktitut – in the same sets of data (expanded) that were used in the study by Lester et al. (2022).

We discovered that a variety of verbalizers and nominalizers were used in variation sets across all stages, with the most frequent ones being used in every stage. Furthermore, variation sets in CDS data involved the use of structures of different morphological complexity: from those with one- and two word class changes, which were present in all stages, to those where the word class changed four times, which were more typical for later stages. We also saw some evidence that the structure of the variation sets became more complex as the stages advanced. These findings suggest that, instead of a decrease in usage of variation sets over time (as was observed in languages with less developed morphology), in morphologically rich languages, variation sets become more sophisticated following the increasing morphological complexity of the caregivers’ speech as their children progress linguistically.

One possible reason for the stable use of variation sets is that, in morphologically rich languages, the difficulties take longer to disperse and it takes longer for children to master the morphological complexity. And, therefore, assuming that variation sets are there to simplify the learning process for the child or make certain morphological features more salient, caregivers would have to use them longer. Another possibility is that, in some cases, variation sets in a language with complex morphology are not a tool for morphological simplification but rather they are a feature of the language itself and are used for rephrasing. In a morphologically complex language, different inflections can be used with the same stem to make different emphasis – not with the purpose of making a certain structure simpler or more salient for the child, but in order to change the emphasis of what one is saying. Similarly, a different morpheme can be put in the midst of a word to provide more information. While in less morphologically complex languages a speaker is likely to reword the sentence, in languages such as Inuktitut, it is easier to change a morpheme than to change the wording completely. Thus, the prolonged use of variation sets in morphologically rich languages might be a spurious correlation and should be investigated in future research.

A possible limitation of the study is that the CDS data we analyzed might not only reflect the linguistic development of the target children, but could also be affected by other factors. During data collection, some of the recording sessions were conducted with people other than mothers and the target children present (e.g., household members, relatives, and the children’s friends). Therefore, while only the mothers’ speech addressed to the target children was analyzed, some of the data could be influenced by other people’s responses and conversations and by the fact that sometimes the mothers addressed several children of different age (e.g., siblings) simultaneously. This limitation is particularly difficult to overcome when the data is comprised of spontaneous naturalistic speech produced in a familiar environment, such as a child’s home, as such an environment can include multiple household members of different generations (see Table 1). Our data comes from both the situations when the mothers and the target children were recorded in the presence of other people and those where they interacted one-on-one.

In our future work, we will continue investigating structures with a word class change in Inuktitut. In particular, we plan to look at such structures in combination with other morphologically complex aspects of Inuktitut such as passive, antipassive and causative (20).

**Table:** 

(20)	*Kiinaujartaatitautuaruvit*?
	kiinaujaq-***taaq-tit-jau***-tuaq-guvit
	money-*acquire-CAUS-PASS*-only-CND.2sS
	‘You are being made to acquire money.’
	(Elijah’s mother addressing Elijah, 2;9)

The use of passive and causative in Inuktitut have been found in previous research to increase in child data by stage, and passive in particular comes in quite early in Inuktitut compared to in many other languages (e.g., Allen and Crago, 1996). Investigating if those aspects by themselves are also facilitated through CDS over time presents another interesting topic for future research.

We see comparing the CDS data to a sample of ADS in Inuktitut as another important step. This would not only pinpoint the differences between the morphology of the two modes in general, but also help better identify the differences of morphological simplification at various stages of children’s linguistic development as ADS would provide the reference point for comparison. Another important aspect of studying CDS that has been addressed in the literature but was beyond the scope of the present study is the alignment in morphology production between CDS and child speech (Crago and Allen, 2001; Ravid et al., 2008; Veneziano and Parisse, 2010). More research should be conducted to investigate whether the input children receive from caregivers influences their production of structures with a word class change and other complex structures in Inuktitut.

The present study contributes in important ways to our understanding of morphological adaptations in CDS. First, the data for the study comes from Inuktitut – a morphologically rich language which presents special challenges for acquisition and, therefore, offers a fruitful field for investigation of the morphosyntax of CDS. Yet, along with many other polysynthetic languages, Inuktitut has not been fully explored in this regard. By studying the use of polysynthesis in Inuktitut CDS, the present study contributes to the relatively small body of research on CDS in morphologically rich languages and on morphology in CDS in general. Second, the results we received support claims of the universality of CDS as a separate mode of speech across languages. Although we did not explicitly conduct a comparative analysis of CDS versus ADS in Inuktitut, the results of this study suggest that, when it comes to morphology, such comparison would reveal a number of differences between the two, placing Inuktitut among the languages that show evidence of CDS as a simplified mode. Finally, to our knowledge, the current study is the first work that concentrates on the nuanced increase of the morphosyntactic complexity in caregivers’ speech and on investigating the use of polysynthetic structures with a word class change in Inuktitut CDS. It demonstrates that Inuktitut-speaking mothers introduce complex polysynthetic structures into their CDS by gradually increasing their frequency and morphological complexity while making their key components - nominalizers and verbalizers – more salient by continuously using them in variation sets, presumably to facilitate their children’s acquisition. These results provide another important insight to the nature of the morphological simplification in child-directed speech and into the larger issue of the nature of input necessary for the first language acquisition.

## Data availability statement

The data analyzed in this study is subject to the following licenses/restrictions: Available only with permission of the researcher. Requests to access these datasets should be directed to (THE LANGUAGE ARCHIVE) [The Language Archive (https://archive.mpi.nl/)] and [THE ACQDIV CORPUS] [UZH – Language, ACQuisition, DIVersity Lab (ACQDIV) – Resources – acqdiv.uzh.ch/en/resources.html].

## Ethics statement

The studies involving human participants were reviewed and approved by Research Ethics Committee, Kativik Ilisarniliriniq. Written informed consent to participate in this study was provided by the participants’ legal guardian/next of kin.

## Author contributions

SA collected and processed the data with crucial assistance of many Inuit transcribers and other research assistants, provided feedback, and helped to shape the manuscript. OJ and SA conceived and planned the study and did relevant morphological coding. OJ carried out the analyses, wrote the first draft of the manuscript, and completed subsequent revisions. Both authors contributed to the article and approved the submitted version.

## Footnotes

1 This simplified mode is used in sign languages as well, and thus should be called *child-directed language*. However, we use the term *child-directed speech* in this article since our study focuses on speech and since the abbreviation *CDS* is well-known.

2 Ages are given as years;months. Thus, 0;11 denotes 0 years and 11 months while 3;6 denotes 3 years and 6 months.

3 The following abbreviations are used in the glosses: 1, first person; 2, second person; 3, third person disjoint; 4, third person coreferential; ABS, absolutive; ALL, allative; ANTP, antipassive; ASP, aspectual; CAUS, causative affix; CND, conditional; CTG, contingent; CTM, contemporative; EMPH, emphatic; EXT, external reference; FUT, future; HAB, habitually; ICM, incontemporative; IMP, imperative; IND, indicative; INCP, inceptive; INT, interrogative; LOC, locative; MOD, modalis; NEG, negative; O, object; PAR, participial; PASS, passive; PAST, past; PEJ, pejorative; PERF, perfective aspect; PL, plural (nominal); POL, politeness affix; PRES, present; PRSP, prospective; S, subject; SG, singular (nominal); d, dual (verbal); p, plural (verbal); s, singular (verbal).

4 The data were originally collected for research on communicative competence (Crago, 1988) and morphosyntactic development in Inuit children (Allen, 1996).

5 If a particular session contained fewer than 15 utterances from the mother, it was not included in our analysis.

6 In all graphs, the dots represent the ‘collapsed’ means for DCPs for each stage.

7 For Inuktitut, Lester et al. (2022) used a subset of the data used in the present study (recordings from months 1, 5, and 9 from the four older children).
