# Supplementary material for: The use of complex structures with a word class change in Inuktitut child-directed speech
Source: Front Psychol. 2022 Oct 7;13:971395. doi: 10.3389/fpsyg.2022.971395 (PMC9585933; doi:10.3389/fpsyg.2022.971395)
Supplement: Supplementary file 1 [file Table_1.pdf]

Appendix. Child-directed speech (CDS) data by stage (determined by children's MLU in morphemes) and by data collection point (DCP): a) mean number of all noun incorporation cases (N→) and b) noun incorporation cases where a noun changes its class more than once (N→V→); c) mean number of all cases of verb-to-noun shifting (V→) and d) cases of verb-to-noun shifting where a verb changes its class more than once (V→N→), per 100 utterances. Means by 100 utterances calculated by dividing the total number of items per DCP by the number of utterances (NU) in that DCP and multiplying by 100.

			per DCP					
Stage_ DCP	Participants' datafiles	Child's age (years; months. days)	Mean Child's MLUm	Mean NU in CDS	Mean N→ per 100 utterances	Mean N→V→ per 100 utterances	Mean V→ per 100 utterances	Mean V→N→ per 100 utterances
1_1	Jini's mother12	0;11.29	1.26	38	2.27		4.54	
1_1	Jini's mother13	1;00.00						
1_2	Jini's mother14	1;04.00	1.27	335	3.28		5.97	2.08
1_2	Jini's mother15	1;04.00						
1_2	Jini's mother16	1;04.00						
1_3	Tumasi's mother22	1;09.12	1.21	185	4.32		3.78	0.54
1_3	Tumasi's mother23	1;09.12						
1_4	Lucasi's mother32	1;08.00	1.43	310	3.87	0.32	2.90	0.64
1_4	Lucasi's mother33	1;08.00						
1_5	Lucasi's mother34	1;11.27	1.44	88	3.40		3.40	
1_5	Lucasi's mother35	1;11.27						
1_5	Lucasi's mother36	1;11.27						
1_6	Lucasi's mother37	2;03.18	1.41	157	3.82		3.82	
1_6	Lucasi's mother38	2;03.18						
1_7	Sarah's mother41	1;03.26	1.09	808	2.35	0.12	4.95	0.12
1_7	Sarah's mother42	1;03.29						
1_7	Sarah's mother43	1;03.29						
1_8	Sarah's mother44	1;07.17	1.31	623	2.24	0.48	1.92	0.80
1_8	Sarah's mother45	1;07.17						
1_8	Sarah's mother46	1;07.17						
2_1	Jini's mother17	1;08.05	1.52	225	2.66	0.88	6.22	2.22
2_1	Jini's mother18	1;08.05						
2_1	Jini's mother19	1;08.05						
2_2	Jini's mother110	2;00.19	1.70	332	6.32	0.60	4.81	2.10
2_2	Jini's mother111	2;00.20						
2_2	Jini's mother112	2;00.20						
2_3	Lucasi's mother310	2;08.12	1.50	124	4.83		2.41	0.80
2_3	Lucasi's mother312	2;08.15						
2_4	Sarah's mother47	1;11.07	1.58	177	7.90	0.56	3.38	1.12
2_4	Sarah's mother48	1;11.07						

2_4	Sarah’s mother49	1;11.07						
2_5	Sarah’s mother411	2;04.05	1.61	185	4.32		0.54	
2_5	Sarah’s mother412	2;04.06						
3_1	Paul’s mother13	2;06.09	2.31	144	8.33		9.72	4.86
3_1	Paul’s mother14	2;06.10						
3_1	Paul’s mother16	2;06.13						
3_2	Elijah’s mother11	2;00.11	2.03	777	11.06	3.21	13.51	3.60
3_3	Louisa’s mother11	2;09.16	2.05	112	7.14			
3_4	Louisa’s mother22	2;10.13	2.20	63	6.34		3.17	3.17
3_5	Louisa’s mother34	2;11.19	2.44	148	10.13		2.02	1.35
3_5	Louisa’s mother38	2;11.19						
3_6	Louisa’s mother41	3;00.12	2.49	245	9.79	2.44	6.53	3.67
3_6	Louisa’s mother42	3;00.14						
3_6	Louisa’s mother43	3;00.14						
3_6	Louisa’s mother48	3;00.15						
3_7	Louisa’s mother51	3;01.15	2.36	124	12.90		7.25	4.03
3_7	Louisa’s mother52	3;01.16						
3_8	Tumasi’s mother25	2;01.11	2.04	89	10.11		5.61	1.12
3_8	Tumasi’s mother26	2;01.11						
3_9	Tumasi’s mother29	2;06.10	2.24	62	4.83		3.22	3.22
4_1	Paul’s mother21	2;07.02	2.86	59	10.16		5.08	3.38
4_2	Paul’s mother32	2;08.09	2.92	160	5.62	0.62	6.25	4.37
4_2	Paul’s mother33	2;08.13						
4_3	Paul’s mother41	2;09.07	2.88	70	5.71		5.71	4.28
4_3	Paul’s mother42	2;09.07						
4_4	Paul’s mother54	2;10.08	2.63	25	4.0	4.0	24.0	8.00
4_5	Elijah’s mother21	2;01.03	2.69	500	8.04	2.8	14.80	5.80
4_5	Elijah’s mother22	2;01.04						
4_6	Elijah’s mother31	2;02.12	2.94	373	9.11	1.87	14.20	6.16
4_7	Elijah’s mother92	2;09.05	2.75	442	9.04	2.26	13.57	3.39
4_8	Lizzi’s mother13	2;06.02	2.96	449	4.45	0.44	6.01	1.11
4_8	Lizzi’s mother14	2;06.03						
4_9	Lizzi’s mother23	2;06.26	2.51	124	10.48	1.61	7.25	1.61
4_9	Lizzi’s mother28	2;06.26						
4_10	Lizzi’s mother34	2;08.05	2.70	421	3.08		4.03	0.47

4_11	Lizzi’s mother41	2;08.27	2.99	285	11.22	1.40	3.15	1.40
4_11	Lizzi’s mother44	2;09.05						
5_1	Paul’s mother63	2;00.10	3.16	124	8.87		15.32	3.22
5_2	Elijah’s mother43	2;03.07	3.48	314	14.33	3.50	15.28	3.50
5_2	Elijah’s mother44	2;03.08						
5_3	Lizzi’s mother51	2;10.03	3.07	546	6.77		4.02	1.83
5_3	Lizzi’s mother52	2;10.04						
5_4	Lizzi’s mother91	3;02.26	3.20	18			5.55	
5_5	Louisa’s mother75	3;03.14	3.44	27	3.70		3.70	3.70
5_6	Louisa’s mother83	3;05.12	3.10	16	6.25			
6_1	Elijah’s mother51	2;04.19	3.70	342	11.69	2.63	14.61	8.77
6_1	Elijah’s mother52	2;04.20						
6_2	Elijah’s mother62	2;06.05	3.52	465	13.54	5.59	16.12	5.16
6_3	Elijah’s mother71	2;07.06	3.64	437	9.83	3.43	16.47	6.63
6_4	Elijah’s mother81	2;08.04	3.75	318	17.29	5.03	14.77	4.08
6_5	Lizzi’s mother61	2;11.27	3.53	169	10.65		10.05	2.95
6_5	Lizzi’s mother63	2;11.29						
6_5	Lizzi’s mother68	2;11.29						
6_6	Lizzi’s mother71	3;00.26	3.51	108	11.11	0.92		
6_7	Lizzi’s mother81	3;01.24	3.93	46	8.69	2.17	4.34	2.17
